# The PPARGC1A Is the Gene Responsible for Thrifty Metabolism Related Metabolic Diseases: A Scoping Review

**DOI:** 10.3390/genes13101894

**Published:** 2022-10-18

**Authors:** Riandini Aisyah, Ahmad Hamim Sadewa, Suryono Yudha Patria, Abdul Wahab

**Affiliations:** 1 Department of Molecular Biology, Faculty of Medicine, Universitas Muhammadiyah Surakarta, Surakarta 57169, Indonesia; 2 Faculty of Medicine, Public Health and Nursing, Universitas Gadjah Mada, Yogyakarta 55281, Indonesia; 3 Department of Biochemistry, Faculty of Medicine, Public Health and Nursing, Universitas Gadjah Mada, Yogyakarta 55281, Indonesia; 4 Division of Pediatric Endocrinology, Department of Pediatrics, Faculty of Medicine, Public Health and Nursing, Universitas Gadjah Mada, Yogyakarta 55281, Indonesia; 5 Department of Biostatistics, Epidemiology and Population Health, Faculty of Medicine, Public Health and Nursing, Universitas Gadjah Mada, Yogyakarta 55281, Indonesia

**Keywords:** thrifty genotype, metabolic syndrome, diabetes mellitus, polymorphism

## Abstract

The “thrifty genotype” hypothesis has thus far described the relationship between specific genes and the population’s resilience to food scarcity circumstances, but its link to the widespread prevalence of genetic diseases and metabolic syndrome has not been adequately mapped. The purpose of the study was to discover genes responsible for thrifty metabolism. A systematic search with keywords was performed for relevant titles. This study used the article’s database published by Pubmed, Proquest, and EBSCO from January, 2009 to September, 2022. Out of 418 papers screened for eligibility, the final evaluation determined that five studies should be included in the analysis. Results indicated that PPARGC1A Gly482Ser led to high BMI in the Tongans population but was unrelated to the onset of type 2 diabetes mellitus, but this was not the case in the Maori population. Significantly differing frequencies of PPAR C1431T and Pro12Ala gene polymorphisms were observed in the Iranian population. GWAS identification of additional genes in Asian and European populations did not produce consistent findings. As a summary, PPARGC1A Gly482Ser addresses as the gene responsible for thrifty metabolism in the Pacific population although some studies show inconsistent results.

## 1. Introduction

The thrifty gene hypothesis was published as a result of the study conducted by JV Neel in 1962 on the function of the thrifty genotype in explaining the epidemiology of type 2 diabetes. This hypothesis explains the genes involved in energy balance regulation and has had a significant impact on our understanding of the genetic history of metabolic diseases [1]. The thrifty gene hypothesis can be understood in two forms: thrifty early and thrifty late. The thrifty early form corresponds more closely to Neel’s perspective, whereas the thrifty late form is the more common view among geneticists. According to the thrifty early hypothesis, risk variants would have been selected for throughout the majority of human evolutionary history, and would therefore be original alleles carried by prehominins and the majority of modern populations [2]. In the past, when people still relied on hunting and gathering for their food, there were both plentiful times and lean times. Those who were more resilient to shifts in food availability were more likely to make it. Since there is always enough to eat in today’s culture, the selective pressures that evolved to help us deal with fluctuations in food availability, including greater energy saving, may no longer be beneficial. Neel’s Thrifty Genotype Hypothesis revolves mostly around this idea. In an investigation of 12 independent, cross-ethnic T2D susceptibility variants, signals of selection pressures on energy metabolism were discovered [3]. Fluctuations in food availability by the influence of seasons and climatic conditions that create pressure for positive selection underlie the emergence of the thrifty gene theory [4]. Thrifty genes are not limited to genes that play a role in regulating energy balance, but also genes related to metabolism and other physiological mechanisms [1]. Positive selection of gene alleles associated with specific traits, such as obesity as a predisposing factor for non-communicable diseases, supports the thrifty gene theory hypothesis in terms of genetic susceptibility [5]. The studies that have been done so far have highlighted whether the main causative factor of metabolic syndrome is glucose intolerance [6].

The Pacific Island population has long been appointed as an example of the thrifty genotype owing to the alarmingly high prevalence of diabetes mellitus type 2, obesity, and other metabolic conditions. It is possible that the limitations of systematic genome-wide association studies in the Pacific population are to blame for the high prevalence of diabetes and obesity risk alleles, while in other world populations such as Asia and Europe, there are limited patterns to support the thrifty gene hypothesis [7]. Diabetes as a multifactorial disease is thought to be a description of the failure of adaptation of gene alleles to current environmental conditions compared to previous conditions, thus, a wide variety of metabolic syndrome manifestations are associated with the response to these changes [8]. Since insulin resistance is currently detrimental and associated with an increased risk of T2D, insulin resistance may have been an adaptive mechanism in the past. A state of insulin resistance would have let the body to respond to physiological stressors such as starvation, inflammation, trauma, and pregnancy by mobilizing glucose energy to maintain important metabolic functions. Priority would have been given to glucose delivery to the brain by reducing glucose uptake by muscle, liver, and adipose tissue. As the human brain is nearly entirely dependent on glucose and a low glucose supply can result in irreversible brain damage, this adaptation process would have been essential to our survival. In the current obesogenic milieu, overnutrition and increasing adiposity can induce a state of persistent low-grade inflammation and hence activate the insulin resistance pathways. This adaptive mechanism that was historically beneficial has turned detrimental in modern society [3].

PPARγ is a transcription factor and is capable of regulating genes, it plays a crucial role in adipogenesis, but has also evolved from thrifty genes. PPARγ has evolved and adapted as a result of both thrifty genes and a challenging environment. Peroxisome proliferator-activated receptor γ coactivator-1α (PGC-1α or PPARGC1A) is the PPARγ transcriptional coactivator and one of the regulatory genes for mitochondrial biogenesis [9]. PPARGC1A activation and expression are influenced by other molecules. Activation of PPARGC1A facilitates the action of SIRT1, of which its overexpression increases the transcriptional activity of PGC-1, whereas a decrease in a molecule involved in glucose and fatty acid metabolism, known as O-linked N modification-acetylglucosamine (O-GlcNAc/OGN), decreases PGC-1 expression [10]. Depending on the acetylation or deacylation status of these proteins, PGC-1 plays a crucial role in modulating and regulating the transcription of regulatory genes involved in mitochondrial biogenesis [11]. PPAR (peroxisome proliferator-activated receptor) is a nuclear receptor superfamily member that plays a role in metabolic pathways and has been reported to be associated with a wide range of disorders, including diabetes, obesity, dyslipidemia, insulin resistance, metabolic syndrome, and coronary heart disease [12,13,14]. Pro12Ala in exon B (rs1801282) and C1431T (rs3856806) in exon 6 are the most prevalent single nucleotide polymorphisms (SNPs) found in the PPAR gene. These two types of polymorphisms are associated with various diseases [14].

Another genetic variation discovered to be associated with type 2 diabetes is genetic variations close to IRS1 (the insulin receptor substrate-1). This gene is a substrate of insulin receptor tyrosine kinase and plays a significant role in the signaling process of insulin and insulin-like growth factor-1; genetic variations in regions adjacent to the IRS1 gene are associated with the occurrence of metabolic disorders [15]. Substitution of glycine to arginine at codon 972 (Gly972Arg) in the IRS1 gene carries a higher risk of insulin resistance. Thus, PPARγ and IRS1 are related to insulin resistance and type 2 diabetes mellitus. IRS1 gene polymorphisms Gly972Arg and PPARγ Pro12Ala are 2 types of polymorphisms associated with predisposing to type 2 diabetes but having opposite effects in their effect on adiponectin levels. Adiponectin is produced by adipose tissue and has been linked to obesity, insulin resistance, type 2 diabetes, and cardiovascular disease. High adiponectin levels found in individuals homozygous for alanine are on PPARGγ (Ala2Ala) and glycine on IRS1 (Gly972Gly) [13]. SNP data on genes were studied using haplotype analysis but this analysis encountered some difficulties related to its underutilization in Genome-Wide Association Studies (GWAS) [16]. The differentiation that occurs in populations can be measured by Wright’s fixation index and Fst which are used to calculate HapMap populations based on the HapMap dataset [15]. The current review aims to determine the genes linked to the thrifty phenotype and the occurrence of the metabolic syndrome as potential candidates for the thrifty gene, while previous studies have focused more on investigating and observing the genes associated with an increased risk of metabolic diseases.

## 2. Materials and Methods

**Figure 1:**
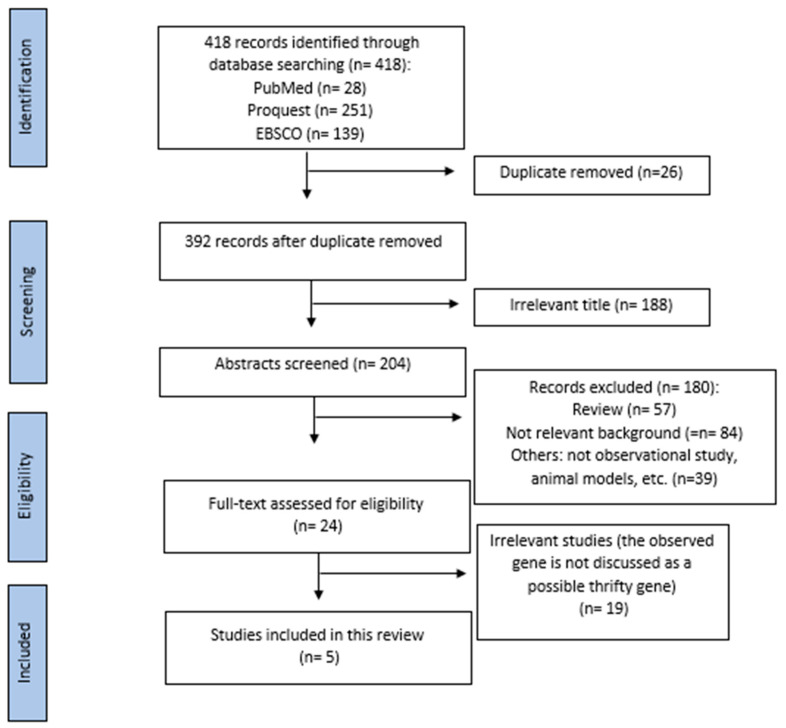
PRISMA diagram of the articles included.

This review was conducted based on the Preferred Reporting Item for the Systematic Reviews and Meta-Analysis (PRISMA). The selected article were then filtered, the irrelevant title of studies were excluded, and further review of the full-text articles assessed for eligibility are taken and reviewed individually by two authors. A total of 418 articles were identified through three databases (Pubmed: 28 articles, EBSCO: 139 articles, Proquest: 251 articles) using the keyword: thrifty gene AND diabetes mellitus between January, 2009–September, 2022. After removing duplicates and excluding irrelevant titles, 204 articles were included for further assessment. We further excluded 180 articles by reading each article’s title and abstract, and the eligibility of the remaining 24 full-text articles was evaluated. Finally, a total of five studies were included in the present study. Data extraction of the studies was done using an Excel table. Figure 1 depicts the flowchart of the articles included in our study.

### Eligibility Criteria

This review included studies from populations in various countries or the HapMap dataset with an observational study design on the genes involved in glucose metabolism in the metabolic syndrome, especially diabetes mellitus with data obtained in the form of genetic variation and distribution. The genotypes obtained from this observational study were then mapped to identify candidate thrifty genes in the population.

## 3. Results

### Study Characteristics

The characteristics of the included observational studies are presented in Table 1. The publishing dates of these papers range from 2011 to 2016. Two of the included articles dealt with the Pacific population, one study was performed in the Iranian population, and the other two studies were performed in the African, European, and Asian populations. One study concluded that PPARGC1A genotype Gly482Ser remains a strong candidate thrifty gene in the Pacific due to the association between Gly482Ser genotypes and BMI in Tongans and the worldwide frequency distribution of the Gly482Ser risk allele [17], while a separate study concluded that this gene should not be considered a candidate thrifty gene locus in Pacific populations, no statistically significant evidence of an association between Gly482Ser PPARGC1A and BMI, type 2 diabetes, or gout was discovered [18]. The study used the HapMap dataset from Europe, Nigeria, China, Japan, and East Asia, suggesting that the rs2943650C non-risk allele of the IRS gene may act as a thrifty allele candidate [15]. The objective of one study was to examine the distribution of two common SNPs of the PPAR gene (C1431T and Pro12Ala) in an Iranian population. The genotypic frequencies of PPAR gene C1431T and Pro12Ala polymorphisms demonstrated significant differences [14]. The remaining study [2] revealed that there was no significant difference between the three populations included for the 64 autosomal index SNPs; thus, the genetic basis of the thrifty gene hypothesis is not supported by only considering the prevalence of type 2 diabetes in current populations.

## 4. Discussion

Neel hypothesized that somehow a “thrifty genotype” may have aided early humans during cycles of feast and famine by boosting the efficiency with which they stored fat when food was plentiful [19]. Another version of this concept is that fat storage as an energy reserve for protracted periods of famine is vulnerable to positive selection [20]. The thrifty gene theory hypothesizes that a tendency to insulin resistance may have saved individuals during times of famine by lowering glucose utilization in tissues such as muscle and boosting its usage by the brain [21]. These ‘efficiency’ fat storers would be greater able to withstand periods of starvation, hence increasing their chances of reproduction. This efficiency only became a drawback when food became routinely accessible. Nevertheless, Neel said nothing about Native Americans. He did not include specific populations in mind, but rather the whole of humanity [19]. In energy ecosystems characterized by periods of abundance and scarcity, accumulation and sedentariness confer a selection advantage. The contemporary obesity epidemic is the result of an inherited propensity to overeat and be sedentary paired with a “thrifty gene” physiological response that allows for the storage of extra energy [22]. Obesity and its associated health problems, according to the thrifty gene theory, are the result of a shift in the types and quantities of foods available [23].

We conducted a review of thrifty genes candidate and genes associated with metabolic diseases, especially type 2 diabetes mellitus, on five selected research articles in the population of the Pacific, Europe, Iran, Africa, and Asia. Table 2 displays the aims and findings of each included study. Diabetes mellitus is a chronic disease that developed when the pancreas failed to produce sufficient insulin or when the body was unable to adequately use the insulin that produces [24]. The thrifty genotype’s metabolic underpinnings may be associated with selective insulin resistance [25]. The risk of type 2 diabetes was significantly elevated by preexisting family history and by chronological decade, but not by living in an urban compared rural setting. It is likely that various wheat strains or even the bread-making process could alter glucose absorption. Environmental chemicals that directly harm pancreatic **β** cells are a second potential [26]. Under the impact of gene regulation, according to the thrifty gene hypothesis, a high carbohydrate diet and a decrease in physical activity promote fat deposition and lead to type 2 diabetes. Possible non-epigenetic contributors to diabetes’s high disease burden include white rice, lifestyle, religion, and their illness model [27].

PPARGC1A Gly482Ser is a potential gene for thriftiness that is found in the Pacific population because observations show that this gene is a strong candidate as a thrifty gene, but this is still a matter of debate due to inconsistent results in the same population. This contradictory conclusion may be influenced by the characteristics of the population obtained. The transcriptional regulatory genes PGC-1 (Peroxisome proliferator-activated receptor coactivator-1) family members play a significant part in the stimulation of mitochondrial biogenesis, oxidative metabolism, peroxisome biogenesis, as well as the metabolism of glucose and fat and are transcriptional co-regulators involved in many metabolic pathways and control of energy metabolism so that it is a potential target for disease prevention and therapeutic interventions related to mitochondrial dysfunction and oxidative metabolism, this is based on the discovery of regulation of PGC-1α expression in several pathological conditions [28,29].

In addition to the functions mentioned above, PPARGC1A is also a specific transcriptional co-activator for the activation of a number of hormone receptors that are essential for the regulation of thermogenesis, cellular respiration, adipogenesis, and oxidative metabolism, as well as agents involved in glucose and lipid metabolism [30]. The expression of the PPARGC1A gene is influenced by polymorphisms, the Gly482Ser variant in this gene is produced by the presence of a base substitution of position 1444 exon 8 on chromosome 4 (4p15.1) where this gene is located, which tends to affect the expression of the PGC-1 and PGC-1 genes in the skeletal muscle. As a candidate polymorphism for the prevalence of type 2 diabetes mellitus and non-alcoholic fatty liver disease, PGC-1 gene polymorphism is a genetic risk factor for diabetes through its role in stimulating mitochondrial biogenesis and enhancing mitochondrial function. PGC-1α expression is lower in diabetic patients than in healthy people, PGC-1 regulates the expression of genes involved in oxidative phosphorylation; therefore, increasing its expression is a crucial factor in the development of glucose metabolism [31].

Myles et al., (2011) examined whether PPARGC1A is a thrifty gene in Pacific populations by testing for an association between Gly482Ser genotypes and BMI in two Pacific populations (Maori and Tongans) and by determining the frequency of the risk allele of the Gly482Ser variant in a global sample of populations. The Gly482Ser variation was shown to be related with BMI in Tongans but not Maori. This risk allele has the highest prevalence in the Pacific. The link between Gly482Ser genotypes and body mass index (BMI) in Tongans and the global frequency distribution of the Gly482Ser risk allele suggest that PPARGC1A remains a putative thrifty gene in Pacific populations [17]. Cadzow et al. (2016) investigated the link between Gly482Ser and T2D, BMI, and gout in a variety of Polynesian populations, as well as Asian, European, and African 1000 Genome Project samples, and reached various conclusions. They conclude that there is insufficient evidence of selection at this locus and that this gene should not be considered as a candidate locus for the thrifty gene in Pacific populations. Possible reasons for high levels of population heterogeneity at this locus and the absence of the derived 482Ser allele in some Melanesian populations include several out-of-Africa migrations by ancestors and subsequent genetic drift during Polynesian colonization. The moderate frequency of the 482Ser allele in modern Western Polynesian populations may be the result of recent hybridization with Melanesian ancestors [18].

Numerous studies have revealed that two PPARγ gene polymorphisms (C1431T and Pro12Ala) are related with several metabolic disorders. Several studies have linked these two common PPARγ polymorphisms to a lowered risk of CAD (coronary artery disease) and metabolic syndrome in different groups and the conclusions have been disputed. Statistical discrepancies in the distribution of two common polymorphisms of the PPARγ gene between the Iranian population and other populations, as reported by Rooki et al., (2013), demonstrated the significance of investigating these SNPs in relation to a number of significant disorders [14].

The thrifty gene hypothesis is based on the alleged adaptation to fluctuations in food availability in the population by these alleles resulting in an increase in BMI, insulin resistance, and type 2 diabetes. This hypothesis also explains the rising incidence of obesity and type 2 diabetes among East Asians [32]. The industrial revolution era has caused changes in nutritional and cultural aspects of the population, and this was followed by a spike in the incidence of metabolic disorders such as type 2 diabetes mellitus [33]. The PPAR gene is required for the regulation of adipogenic differentiation and glucose homeostasis located on chromosome 3p25 and by alternative mRNA splicing produces three different isoforms (PPARγ1, PPARγ2, and PPARγ3) and is found mainly in adipose tissue [34].

IRS1 (insulin receptor substrate-1) is an important gene associated with diabetes because it is a substrate for insulin receptor tyrosine kinase and plays a crucial role in insulin signaling, insulin-like growth factor-1 signaling and regulates insulin secretion by **β** cells in the pancreas. Autocrine insulin signaling pathways, including insulin receptor phosphorylation, tyrosine phosphorylation on IRS1, and PI3-kinase activation, stimulate glucose-stimulated insulin secretion. Genes associated with type 2 diabetes mellitus, insulin resistance, hyperinsulinemia, adiposity, and CAD are located adjacent to the IRS1 gene, which possesses common genetic variation [15]. The likelihood of a positive effect of allele A on the investigated polymorphisms IRS1 -rs10498210 G/A and CCR5-59029 A/G elevates the chance of developing type 2 diabetes [35]. Results obtained by Yoshiuchi et al., (2013) in European, Nigerian, Chinese, Japanese, and East Asian populations showed that the IRS gene in the SNP associated with insulin resistance (rs2943650) exhibited high Fst values (a measure of population differentiation), indicating that the rs2943650C non-risk allele might be candidate thrifty allele in that population.

Trying to assess the selection of genes may be both complicated and challenging, but these attempts to determine selection in T2DM-associated genes could be extremely beneficial for clinical medicine and diabetes clinics in avoiding the onset of diabetes and managing diabetes patients using genotypes of preferred T2DM genes. The rate of T2DM is increasing globally, and it can result in the development of critical conditions. Important genes may have been the target of natural selection and become more differentiated throughout the past 10,000 years of human history, allowing people to thrive and live in environments with different regions, temperatures, and food supplies. The important selected genes may therefore leave historical traces in our present genome [15]. Currently, a significantly larger number of index SNPs and other tools for detecting positive selection are accessible. Therefore, Ayub et al., (2014) were able to conduct a thorough reassessment of this question, and their findings were consistent with those of many previous studies, notably that there was no significant enhancement for positive selection on the loci as a whole, but there was evidence for positive selection at a limited number of individual loci.

## 5. Conclusions

Differences in the prevalence of metabolic diseases in the population resulted from the frequency of polymorphisms and different risk alleles between populations. The existence of the thrifty genotype hypothesis provides an explanation for the high prevalence of metabolic diseases. Genetic variations in genes related to metabolic diseases, especially diabetes mellitus, are considered to be candidates for thrifty genes in the population. Some of these gene variants are the Gly482Ser variant in PPARGC1A, PPARγ gene C1431T and Pro12Ala, IRS1 gene variant rs2943650C non-risk allele. PPARGC1A Gly482Ser is considered a candidate for the thrifty gene in the Pacific population, although identification results for these genes in other populations are inconsistent.

## Figures and Tables

**Table 1:** Characteristics of selected studies.

First Author, Published Year	Population	Study Design	Number of Subjects	Outcome (Thrifty Gene Candidate)
Myles S., 2011 [17]	Tongans and Maori	Observational	294 individuals and the allele frequencies of the 482Ser risk allele across 58 world-wide populations	PPARGC1A genotype Gly482Ser remains a strong candidate for a gene associated with thriftiness in the Pacific.
Yoshiuchi I., 2012 [15]	European, Nigeria, China, Japan, East Asia	Observational	HapMap dataset	High Fst values were observed for 1 CAD-associated SNP (rs2943634) and 1 adiposity- and insulin resistance-associated SNP (rs2943650) of the IRS gene. The rs2943650C risk-free allele may be a thrifty allele.
Rooki H., 2013 [14]	Iranian	Observational	160 healthy Iranian individuals	Significant differences were discovered in the PPAR gene C1431T and Pro12Ala polymorphism frequencies between the Iranian population and others
Ayub Q., 2014 [2]	African, European, Asian	Observational	65 (type 2 diabetes mellitus) index SNPs	The distributions of the pairwise Fst values for the 64 autosomal index SNPs between the three continental groups and the matched controls and stratification groups are not significantly different. However, failure to detect signal across all 64 loci does not necessarily rule out the presence of signals at some loci when tested separately: - Five index SNPs (rs340874, rs3923113, rs6780569, rs1470579, and rs1552224) at five GWAS loci (near PROX1 [MIM 601546], GRB14 [MIM 601524], UEB2E2 [MIM 602163], IGF2BP2 [MIM 608289], and ARAP1 [MIM 606646]) in African populations - Three index SNPs (rs340874, rs1531343, and rs8042680) at three GWAS loci (those near PROX1, HMGA2 [MIM 600698], and PRC1 [MIM 603484]) in European populations - Nine index SNPs (rs10923931, rs11899863, rs7578597, rs3923113, rs10010131, rs1801214, rs896854, rs7903146, and rs8042680) at seven GWAS loci (NOTCH2, THADA [MIM 611800], GRB14, WFS1 [MIM 606201], TP53INP1 [MIM 606185], TCF7L2, and PRC1) in East Asian populations showed significant signals.
Cadzow M., 2016 [18]	Polynesian	Observational	All individuals in the Māori AXIOM and Samoan OMNIexpress datasets. Five additional populations were obtained from the 1000 Genomes Project phase 3 data release.	Gly482Ser PPARGC1A was not associated with BMI, type 2 diabetes, or gout, according to statistical analysis. This gene should not be regarded a candidate thrifty gene locus in Pacific populations

**Table 2:** Research objectives and results of included studies.

No	Title	Objective	Statistical Analysis	Result
1.	Testing the thrifty gene hypothesis: the Gly482Ser variant in PPARGC1A is associated with BMI in Tongans [17]	Test the thrifty gene hypothesis in Pacific populations by examining the relationship between Gly482Ser genotypes and body mass index (BMI) in Maori and Tongans and by comparing the frequency of the 482Ser risk allele in Pacific populations to populations worldwide.	Performing ANOVA and multiple regression in R, evidence of association between body mass index, age, sex, population, and genotype was investigated.	In Tongans, but not Maori, the Gly482Ser variant is related with BMI. The frequency of the 482Ser risk allele is highest in the Pacific, according to a study of 58 populations from around the world. The association between Gly482Ser genotypes and body mass index (BMI) in Tongans and the global frequency distribution of the Gly482Ser risk allele indicates that PPARGC1A represents a candidate thrifty gene in Pacific populations.
2.	Evidence of selection at insulin receptor substrate-1 (IRS1) gene loci [15]	Discover evidence of selection at the IRS1 gene loci using the HapMap population data.	Wright's Fst, the LRH test, and the iHS test were used to find selection at the IRS1 loci in the HapMap population.	Fst analysis: high Fst values in 1 CAD-associated SNP (rs2943634:0.387) and 1 adiposity- and insulin resistance-associated SNP (rs2943650:0.384). LRH test: the core haplotype with 1 T2DM risk allele (rs7578326A) in the East Asian populations showed evidence of selection (REHH percentile, 99.9). The rs7578326A risk allele of the IRS1 loci is apparently associated with T2DM. The core haplotype with 1 CAD risk allele (rs2943634C) in the East Asian populations revealed evidence of selection (REHH percentile, 99.9). iHS test: the highest [iHS] score of the SNP rs12694705 was 3.13 in the CEU population, the highest [iHS] score of the SNP rs16867016 was 3.19 in the YRI population, and the highest [iHS] score of the SNP rs13395556 was 2.94 in the East Asian.
3.	Distribution and genotype frequency of the C1431T and pro12ala polymorphisms of the peroxisome proliferator activator receptor γ gene in an Iranian population [14]	Investigate the frequency of two common single nucleotide polymorphisms of the PPARγ gene (C1431T and Pro12Ala) in an Iranian population	Chi square (2) or Fisher's exact test was used to assess the statistical significance of differences in genotypic distributions between populations.	The genotypic frequency distribution of the C1431T PPAR polymorphism was 0.869 for CC, 0.119 for CT, and 0.013 for TT, whereas the allelic frequency distribution was 0.93 for C and 0.07 for T, respectively. For another Pro12Ala variant of PPAR, the genotypic distributions and allelic frequencies were as follows: 0.813 for CC, 0.181 for CG, 0.06 for GG, 0.903 for C, and 0.097 for G.
4.	Revisiting the Thrifty Gene Hypothesis via 65 Loci Associated with Susceptibility to Type 2 Diabetes [2]	Investigated the thrifty gene hypothesis.	Site frequency spectrum-based analyses, CMS analysis, haplotype diversity, pairwise FST.	No evidence to support the thrifty early hypothesis, no support for the thrifty late hypothesis, and individual genes showing nominal evidence of positive selection.
5.	Lack of direct evidence for natural selection at the candidate thrifty gene locus, PPARGC1A [18]	To explicitly test the hypothesis that the PPARGC1A locus, specifically the Gly482Ser substitution, was susceptible to natural selection in the ancestors of modern Polynesian populations.	To identify associations between BMI and rs8192678 genotype, single marker linear regressions were performed using the R statistical software environment. A logistic regression was undertaken for T2D and gout affection status. Pairwise FST was estimated for all populations for rs8192678. FST between sample sets, Tajima’s D, Fay and Wu’s H, and integrated haplotype score (iHS, for individual populations, and cross population haplotype homozygosity (XP-EHH, to assess selection between populations. A customized analytical pipeline was performed to calculate these statistics	Tests for association between BMI and rs8192678 genotype did not show any statistically significant association for any of the gene action models, no relationship was observed between rs8192678 genotype and either gout affection or T2D status, for any of the modes of genetic action, and also no statistically significant associations detected for the meta-analysis of the Polynesian populations between BMI, or gout affection, or T2D status and rs8192678 under any of the mechanism of genetic action.

## Data Availability

Not applicable.
